# Aspect-Aware Target Detection and Localization by Wireless Sensor Networks

**DOI:** 10.3390/s18092810

**Published:** 2018-08-25

**Authors:** Li Hu, Shilian Wang, Eryang Zhang

**Affiliations:** College of Electronic Science, National University of Defense Technology, Changsha 410073, China; huli09@nudt.edu.cn (L.H.); zhangeryangnudt@163.com (E.Z.)

**Keywords:** aspect dependent target, wireless sensor network (WSN), joint detection and localization, generalized likelihood ratio test (GLRT), maximum likelihood estimate (MLE), observation quantization

## Abstract

This paper considers the active detection of a stealth target with aspect dependent reflection (e.g., submarine, aircraft, etc.) using wireless sensor networks (WSNs). When the target is detected, its localization is also of interest. Due to stringent bandwidth and energy constraints, sensor observations are quantized into few-bit data individually and then transmitted to a fusion center (FC), where a generalized likelihood ratio test (GLRT) detector is employed to achieve target detection and maximum likelihood estimation of the target location simultaneously. In this context, we first develop a GLRT detector using one-bit quantized data which is shown to outperform the typical counting rule and the detection scheme based on the scan statistic. We further propose a GLRT detector based on adaptive multi-bit quantization, where the sensor observations are more precisely quantized, and the quantized data can be efficiently transmitted to the FC. The Cramer-Rao lower bound (CRLB) of the estimate of target location is also derived for the GLRT detector. The simulation results show that the proposed GLRT detector with adaptive 2-bit quantization achieves much better performance than the GLRT based on one-bit quantization, at the cost of only a minor increase in communication overhead.

## 1. Introduction

Recently, wireless sensor networks (WSNs) have received considerable attention due to their applicability to reconnaissance, surveillance, security, and environmental monitoring [1,2]. A typical WSN consists of a large number of low-cost battery-powered devices and usually has very stringent energy and bandwidth constraints [3].

The detection of specific events (targets, transmitters, etc.) is one of the main tasks for a WSN [4]. In a detection system based on WSN, centralized and decentralized frameworks are two typical setups for information transmission from sensors to the fusion center (FC) which is in charge of making the final decision [5]. In a centralized framework, the FC has full knowledge of all sensor observations, which results in optimal performance. However, the centralized setup amounts to instantaneous high-precision communication between sensors and the FC [6], which is impractical for WSNs due to the stringent bandwidth and energy constraints. To address this issue, decentralized schemes that allow sensors to transmit a few condensed information bits to the FC, are of significant interest. For decentralized target detection by a WSN, one of the key problems is how to quantize the sensor observations. Some one-bit quantization [7] and multi-level quantization schemes [8,9] have been studied for decentralized detection. However, these quantization schemes only work under the assumptions of Gaussian noise and a single unknown parameter.

When detecting a non-cooperative target (or signal) with unknown parameters in a WSN, a natural strategy is the so-called counting rule test, namely, the FC counts the number of local on-off detections and compares it with a threshold [10]. The generalized likelihood ratio test (GLRT) detector, which is obtained by substituting the unknown parameters with their maximum likelihood estimates (MLEs), is also commonly employed at the FC to make a final decision [11]. A GLRT detector based on one-bit quantized data was studied in refs. [7,12] to detect (i) an unknown scalar deterministic signal, and (ii) a random Gaussian signal with an unknown variance. GLRT and its computationally simpler alternatives were considered in [13,14,15,16,17] to detect a non-cooperative target with spatially dependent emission. In particular, GLRT fusion was derived in the context of detecting a target emitting a known signal at an unknown location [13], and detecting an unknown transmitter with an unknown location [14]. In the latter case, a complicated grid search on both the target location and emitted power domains may be required for implementation. To reduce the computational complexity, a generalized Rao test was developed in ref. [15]. Different from refs. [13,14,15] that treated the target emission as a deterministic signal, the emitted signal was presumed to be unknown and fluctuating in refs. [16,17], where generalized forms of locally optimum detectors were proposed as computationally simpler alternatives to the GLRT. While these works dealt with targets that emit or reflect a isotropic signal, the detection of a target with aspect dependent reflection is still under study.

A few works have addressed the localization [18,19] or detection [20] of a target with aspect dependent reflection in a WSN. More specifically, a model accounting for the aspect dependence of the reflected signal from a underwater target was utilized for the MLE of a submarine’s location in ref. [18]. In ref. [19], a scenario involving the location of low-visibility ground or air targets using multiple unmanned autonomous vehicles and a large number of distributed sensors, was considered. Later, in ref. [20], Song et al., studied the underwater target detection with a barrier sensor network. There, the aspect and distance dependence of the acoustic reflection signal of underwater targets were considered, and binary local decisions were fused in the FC via scan statistic to form a global decision. However, such a scan statistic-based detection schemes have obvious limitations, namely, numerous sensors are required to make a reliable decision, and target localization can not be achieved.

In this paper, we consider the detection and localization of a target with aspect dependent reflection. The detection and localization tasks are accomplished by the GLRT detector, where the MLE of target location is given as a by-product of the GLRT once the target is detected. The main contributions can be summarized as follows:

- A GLRT detector based on one-bit quantized data is developed. If the target is detected, the MLE of the target location is given as a by-product of the GLRT detector. The differential evolution (DE) algorithm is introduced to solve the MLE. It is shown that the GLRT detector with one-bit quantization outperforms the existing detection scheme based on scan statistic [20].
- A GLRT detector based on adaptive multi-bit quantization is proposed to further improve the detection and localization performance. The proposed adaptive quantizer achieves higher quantization precision; meanwhile, its yielding data has a greatly reduced communication burden compared with the typical multi-bit quantization scheme. The Cramer–Rao lower bound (CRLB) of the MLE is also derived.

The rest of this paper is organized as follows. In Section 2, we briefly introduce the active target detection and localization model where the reflected signal of the target is aspect and distance dependent. Then, the GLRT detector based on one-bit quantized data is studied in Section 3. In Section 4, we propose a GLRT detector based on adaptive multi-bit quantization, together with its CRLB derivation and communication overhead analysis. Numerical results and comparisons are provided in Section 5. Finally, Section 6 concludes the paper.

## 2. Problem Statement

**Figure 1:**
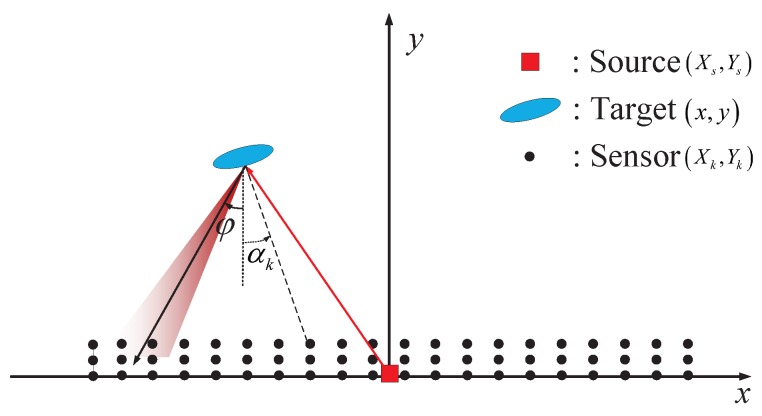
Low-visibility target detection by a sensor network where the reflected signal of the target is aspect and distance dependent.

Consider an active detection system that consists of an active source, *K* passive sensors, and an FC. As illustrated in Figure 1, the source first sends out a probing signal to the surveillance area for target detection as well as to all the sensors to activate them from sleep mode. If a target exists, the reflected signal from the target is concentrated within a narrow angle. In addition to such an aspect dependence, the reflected signal is also distance dependent due to free-space attenuation. The observed information is then transmitted from sensors to the FC where the global decision is made.

### 2.1. Observing the Reflected Signal at Sensors

Since the waveform of the probing signal is known to sensors, a matched filter can be applied to preprocess the received signal, thus yielding the observation $r_{k}$, where k=1,2,…,*K* is the sensor index. Assume that sensor noises are independent and identically distributed zero-mean complex Gaussian variables with a variance of $\sigma^{2}$. In the absence of a target ($H_{0}$), $r_{k}$ is exponentially distributed, and has a probability density function (pdf) of [20,21]

(1) $$\begin{array}{c} f_{0}^{k}\left(r_{k}\right)=\frac{1}{2\eta \sigma^{2}}\exp\left(-\frac{r_{k}}{2\eta \sigma^{2}}\right), \end{array}$$

where η is the known waveform energy. In the presence of a target ($H_{1}$), the received signal at the sensors is a combination of the reflected signal and the noise. The energy of the reflected signal depends on the sensor–target geometry and the target aspect, which is parameterized by $\epsilon_{k}$. Assuming a Rayleigh fading signal model, the pdf of $r_{k}$ can be written as [20,21]

(2) $$\begin{array}{ccc} f_{1}^{k}\left(r_{k}\right) & = & \frac{1}{2\eta \sigma^{2}+2\eta \epsilon_{k}^{2}}\exp\left(-\frac{r_{k}}{2\eta \sigma^{2}+2\eta \epsilon_{k}^{2}}\right)\\ & ≜ & \frac{1}{2\eta \sigma^{2}\left(1+\rho^{k}\right)}\exp\left(-\frac{r_{k}}{2\eta \sigma^{2}\left(1+\rho^{k}\right)}\right), \end{array}$$

where the definition $\rho^{k}≜\frac{\eta \epsilon_{k}^{2}}{\sigma^{2}}$ is employed. In accordance with refs. [18,19,20], $\rho^{k}$ is specified by

(3) $$\begin{array}{c} \rho^{k}=C_{0}g_{1}\left(d_{k}\right)g_{2}\left(\alpha_{k},\varphi \right), \end{array}$$

where $C_{0}$ is a constant, $g_{1}\left(\cdot \right)$ specifies the power loss caused by the propagation distance, and $g_{2}\left(\cdot \right)$ describes the aspect dependence of the reflected signal. More specifically, $d_{k}$ denotes the propagation distance of the signal from the source to the *k*-th sensor via target reflection, i.e.,

(4) $$\begin{array}{c} d_{k}=\sqrt{{\left(x-X_{s}\right)}^{2}+{\left(y-Y_{s}\right)}^{2}}+\sqrt{{\left(x-X_{k}\right)}^{2}+{\left(y-Y_{k}\right)}^{2}}, \end{array}$$

with (x,y), $\left(X_{s},Y_{s}\right)$ and $\left(X_{k},Y_{k}\right)$ being locations of the target, the active source, and the *k*-th sensor, respectively; $\alpha_{k}$ is the angle from the *k*-th sensor to the target:

(5) $$\begin{array}{c} \alpha_{k}=\arctan\left(-\frac{x-X_{k}}{y-Y_{k}}\right), \end{array}$$

and φ is the principal reflection angle of the target which implies the aspect dependence of the target reflection.

Next, we define the attenuation funtion ($g_{1}\left(d_{k}\right)$) and the aspect dependence ($g_{2}\left(\alpha_{k},\varphi \right)$). Assuming free-space attenuation, we have [19]

(6) $$\begin{array}{c} g_{1}\left(d_{k}\right)={\left(d_{k}\right)}^{-\beta }, \end{array}$$

where β=1 corresponds to cylindrical spreading, β=2 is spherical spreading, and sometimes, larger values of β are used to model loss due to shadowing, e.g., β=3.5. In this work, we set β=1, and the corresponding development can be easily extended to other types of attenuation.

On the other hand, the aspect dependent reflection gain is modeled by the following Butterworth filter [18,19,20]:

(7) $$\begin{array}{c} g_{2}\left(\alpha_{k},\varphi \right)=\frac{1}{1+{\left(\frac{\alpha_{k}-\varphi }{\Phi }\right)}^{2\Omega }}, \end{array}$$

where Ω denotes the filter order, and 2Φ is the 3 dB bandwidth. Although the exact function ($g_{2}\left(\cdot \right)$) might be different from (7) under specific circumstances, the following analysis can be similarly performed for other aspect dependent reflection models as long as its closed-form expression is available.

In the following analysis, we assume perfect knowledge of the model parameters $\sigma^{2},C_{0},\Phi$ and Ω. In practice, these values could be estimated based on training data. In addition, sensor positions are assumed to be known to the FC, since they can be estimated periodically during network maintenance (see, e.g., refs. [22,23], and references therein). However, under hypothesis $H_{1}$, the target’s position ((x,y)) and the principal reflection angle (φ) remain unknown.

### 2.2. Data Transmission and Global Decision Making

After making observations, the sensors send their local information to the FC which makes a final decision on whether the target exists or not. To meet the stringent energy and bandwidth constraints in WSNs, sensor measurements are usually quantized into few-bit data individually before transmission. The design of a quantizer is one of the focuses of this work.

At the FC, a composite binary hypothesis testing problem is addressed due to the unknown target parameters. GLRT solutions, which achieve target detection and localization simultaneously, are also of interest here.

## 3. Target Detection and Localization by GLRT Utilizing One-Bit Quantized Data

We start with the scenario where sensors only transmit one-bit of information to the FC. Faced with a composite hypothesis testing problem, the FC employs the GLRT approach which is obtained by substituting the unknown parameters with their MLEs into a general likelihood ratio test. The DE algorithm is employed to solve the MLE of unknown target parameters since no closed-form solutions are available for the MLE. The CRLB of the MLE is also given for evaluation of the target localization performance.

### 3.1. Derivation of GLRT Detector

The optimal one-bit quantization at each sensor is known to be a thresholding rule of the likelihood ratio [15]. However, it is impractical to compute the likelihood ratio at each sensor due to the unknown target parameters. A natural one-bit quantization strategy is to compare each sensor’s observation with a preset threshold Δ [18,20], which produces the output ${\left\{b_{k}\right\}}_{k=1}^{K}$ as follows:

(8) $$\begin{array}{c} b_{k}=\{\begin{array}{cc} 1, & \mathrm{if}\;r_{k}\geq \Delta ,\\ 0, & \mathrm{otherwise}, \end{array},\;k=1,2,\cdots ,K. \end{array}$$

We first derive the distribution of the quantizer output, ${\left\{b_{k}\right\}}_{k=1}^{K}$. In accordance with refs. (1) and (2), the probabilities that $b_{k}$ takes a value of 1 under hypotheses $H_{0}$ and $H_{1}$ are respectively given by

(9) $$\begin{array}{c} P_{0}≜\mathrm{Pr}\left(b_{k}=1|H_{0}\right)=\int_{0}^{\Delta }f_{0}^{k}\left(r_{k}\right)dr_{k}=\exp\left(-\frac{\Delta }{2\eta \sigma^{2}}\right), \end{array}$$

and

(10) $$\begin{array}{c} P_{1,\theta }^{k}≜\mathrm{Pr}\left(b_{k}=1|H_{1}\right)=\int_{0}^{\Delta }f_{1,\theta }^{k}\left(r_{k}\right)dr_{k}=\exp\left(-\frac{\Delta }{2\eta \sigma^{2}\left(1+\rho_{\theta }^{k}\right)}\right), \end{array}$$

where θ denotes the vector of all unknown parameters, i.e., θ≜[x,y,φ], and $f_{1,\theta }^{k}\left(r_{k}\right)$ is the pdf of the received signal under $H_{1}$ with specific θ. Also, $\rho_{1,\theta }^{k}$ is the value of $\rho^{k}$ with a given θ. Using the notations $P_{0}$ and $P_{1,\theta }^{k}$, the probability mass functions (pmfs) of $b_{k}$ under hypotheses $H_{0}$ and $H_{1}$ are given as follows, respectively:

(11) $$\begin{array}{c} h_{0}^{k}\left(b_{k}\right)={\left(P_{0}\right)}^{b_{k}}{\left(1-P_{0}\right)}^{1-b_{k}}, \end{array}$$

and

(12) $$\begin{array}{c} h_{1,\theta }^{k}\left(b_{k}\right)={\left(P_{1,\theta }^{k}\right)}^{b_{k}}{\left(1-P_{1,\theta }^{k}\right)}^{1-b_{k}}. \end{array}$$

Assuming that all the binary data b $≜[b_{1},b_{2},\cdots ,b_{K}]$ can be received by the FC without errors, the FC is faced with the following composite binary hypothesis testing problem:

(13) $$\begin{array}{c} \left\{ \begin{array}{cc} H_{0}: & b_{k}∼h_{0}^{k}\left(b_{k}\right),\;k=1,2,\cdots ,K,\\ H_{1}: & b_{k}∼h_{1,\theta }^{k}\left(b_{k}\right),\;k=1,2,\cdots ,K, \end{array}\right. \end{array}$$

where ∼ denotes “distributed according to”. A natural approach to solve the composite hypothesis testing problem above is to replace the unknown parameters with their MLEs [11], which yields the GLRT detector,

(14) $$\begin{array}{c} \left\{\tilde{T}\left(b\right)≜\log\frac{\max_{\theta }\prod_{k=1}^{K}h_{1,\theta }^{k}\left(b_{k}\right)}{\prod_{k=1}^{K}h_{0}^{k}\left(b_{k}\right)}\right\}\overset{H_{1}}{\underset{H_{0}}{≷}}\lambda , \end{array}$$

where λ is a prescribed decision threshold to satisfy certain error probabilities. After substituting (11) and (12) into (14), the generalized log-likelihood ratio (GLLR) $\tilde{T}\left(b\right)$ can be written as:

(15) $$\begin{array}{ccc} \tilde{T}\left(b\right) & = & \max_{\theta }\sum_{k=1}^{K}\log h_{1,\theta }^{k}\left(b_{k}\right)-\sum_{k=1}^{K}\log h_{0}^{k}\left(b_{k}\right)\\ & = & \sum_{k=1}^{K}\left[b_{k}\log\frac{P_{1,\tilde{\theta }}^{k}}{P_{0}}+\left(1-b_{k}\right)\log\frac{1-P_{1,\tilde{\theta }}^{k}}{1-P_{0}}\right]. \end{array}$$

In ref. (15), $\tilde{\theta }$ denotes the MLE of the unknown parameter vector θ, which is obtained by maximizing the log-likelihood function of θ, i.e.,

(16) $$\begin{array}{c} \tilde{\theta }=\arg\max_{\theta }{\tilde{L}}_{b}\left(\theta \right), \end{array}$$

where

(17) $$\begin{array}{c} {\tilde{L}}_{b}\left(\theta \right)=\sum_{k=1}^{K}\left[b_{k}\log P_{1,\theta }^{k}+\left(1-b_{k}\right)\log\left(1-P_{1,\theta }^{k}\right)\right]. \end{array}$$

Clearly, if a target is detected, $\tilde{\theta }$ provides the desired estimation of the target location.

### 3.2. Solve the MLE with the Differential Evolution Algorithm

Note that no closed-form solution can be found for (16) due to the complicated objective function. Conventional optimization methods, such as the gradient decent method, to a great extent, rely on the assumption of the objective function’s characteristics (e.g., concavity, convexity, and monotonicity). However, it is difficult to check the concavity of the objective function in (17). Furthermore, it is a subtle task to tune the parameters of conventional optimization methods for high-quality solutions. On the other hand, the DE algorithm [24] addresses these issues properly. Thus, we resort to the DE algorithm to solve (16).

The DE algorithm imitates the biological evolution and can generate a good solution that maximizes (or minimizes) the objective function via the iterative process of reproduction and selection [25]. Specifically, the DE first randomly initializes the population to cover the entire search space uniformly. The individuals of the population are then perturbed and combined by applying mutation and crossover operators to produce new candidates. The better individuals among the current population and the newly produced candidates are selected, constituting the next generation. The mutation, crossover, and selection procedures are repeated until the stopping criterion is satisfied. Then, the best individual among the last generation is chosen as the final solution.

In this work, the detailed steps used to solve the MLE by the DE algorithm were as follows. First, given the size of the population ($N_{p}$), the scalar number (*F*), the crossover rate ($C_{r}$), the maximum number of generations ($G_{\max}$), the maximum bounds of the parameters were $\theta_{\max}=\left[x_{\max},y_{\max},\varphi_{\max}\right]$ and the minimum bounds were $\theta_{\min}=\left[x_{\min},y_{\min},\varphi_{\min}\right]$.

1. **Initialization.** The generation number was set to G=0 and $N_{p}$ individuals were randomly initialized with their goal vectors ($\theta_{i,G}=\left[\theta_{1,i,G},\theta_{2,i,G},\theta_{3,i,G}\right]$) uniformly drawn from the search space $\left(\theta_{\min},\theta_{\max}\right)$, where $i=1,2,\cdots ,N_{p}$.
2. **Generation Evolution.** While the current generation count ($G<G_{\max}$) was occurring, **Steps** 2.1–2.3 were performed for each individual *i*, and then G=G+1 was set.
  **Step** 2.1: ***Mutation.*** Create a donor vector $\vartheta_{i,G}=\left[\vartheta_{1,i,G},\vartheta_{2,i,G},\vartheta_{3,i,G}\right]$ for the *i*-th goal vector following the differential mutation scheme:
  (18) $$\begin{array}{c} \vartheta_{i,G}=\theta_{\gamma_{1}^{i},G}+F\left(\theta_{\gamma_{2}^{i},G}-\theta_{\gamma_{3}^{i},G}\right). \end{array}$$
  The indices $\gamma_{1}^{i}$, $\gamma_{2}^{i}$, and $\gamma_{3}^{i}$ are different integers randomly chosen from $\left\{1,2,\cdots ,N_{p}\right\}$, which are also different from the current goal vector index (*i*).
  **Step** 2.2: ***Crossover.*** On the basis of goal vector $\theta_{i,G}$ and donor vector $\vartheta_{i,G}$, generate a trial vector $\mu_{i,G}=\left[\mu_{1,i,G},\mu_{2,i,G},\mu_{3,i,G}\right]$ by performing the following crossover operation on each of the three components:
  (19) $$\begin{array}{c} \mu_{j,i,G}=\{\begin{array}{cc} \vartheta_{j,i,G} & \mathrm{if}\;({\mathrm{rand}}_{i,j}\leq C_{r}\;\mathrm{or}\;j=j_{\mathrm{rand}}),\\ \theta_{j,i,G} & \mathrm{otherwise}, \end{array},\;j=1,2,3, \end{array}$$
  where ${\mathrm{rand}}_{i,j}$ is a uniformly distributed random number within (0,1), and $j_{\mathrm{rand}}\in \left\{1,2,3\right\}$ is a randomly chosen index ensuring that $\mu_{i,G}$ gets at least one component from $\vartheta_{i,G}$.
  **Step** 2.3: ***Selection.*** Determine whether the goal vector $\theta_{i,G}$ or the trial vector $\mu_{i,G}$ survives to the next generation by comparing their objective function values, which are respectively calculated by substituting $\theta_{i,G}$ and $\mu_{i,G}$ into (17), i.e.,
  (20) $$\begin{array}{c} \theta_{i,G+1}=\{\begin{array}{cc} \mu_{i,G} & \mathrm{if}\;{\hat{L}}_{b}\left(\mu_{i,G}\right)\geq {\hat{L}}_{b}\left(\theta_{i,G}\right),\\ \theta_{i,G} & \mathrm{otherwise}. \end{array} \end{array}$$
3. **Termination.** The iteration in Step 2 stops at the $G_{\max}$-th generation. Among the $N_{p}$ goal vectors of the $G_{\max}$-th generation, the goal vector that yields the largest objective function value is chosen as the final solution.

We note that the runtime-complexity of solving the MLE by the DE method is $O(3\cdot N_{p}\cdot G_{\max})$, since, in each generation of the DE, a loop over $N_{p}$ is performed, containing a loop over the three components of θ in the mutation and crossover operations.

### 3.3. Discussion

It is well known that false alarm probability and detection probability are performance indicators of any given detection rule and are defined as

(21) $$\begin{array}{c} P_{fa}=\mathrm{Pr}\left\{\tilde{T}\left(b\right)\geq \lambda |H_{0}\right\},\mathrm{and}\;P_{d}=\mathrm{Pr}\left\{\tilde{T}\left(b\right)\geq \lambda |H_{1}\right\}. \end{array}$$

However, $P_{fa}$ and $P_{d}$ cannot be analytically derived since the conditional pdfs of $P(\tilde{T}\left(b\right)|H_{0})$, and $P(\tilde{T}\left(b\right)|H_{1})$ are unavailable. Nevertheless, the simulation results under target absence hypothesis may help the designer choose a threshold (λ) to form a constant false alarm rate detector. $P_{d}$ is also evaluated by simulations, which is acceptable since $P_{d}$ is target dependent in any case.

If a target is detected, $\tilde{\theta }$ provides the MLEs of the target location and the principal reflection angle. The theoretical performance limits of the MLEs are given by the CRLB matrix. It is worth mentioning that the CRLB of the MLE was derived for underwater target localization in ref. [18], and that analysis is also applicable here. Therefore, the Fisher information matrix (FIM), which is employed to characterize the CRLB, is a 3×3 matrix with the $\left(i,j\right)$-th element given by ref. [18]

(22) $$\begin{array}{c} {\left[\;\tilde{I}\;\right]}_{i,j}=\sum_{k=1}^{K}\left\{\frac{\Delta^{2}P_{1,\theta }^{k}}{4\eta^{2}\sigma^{4}{\left(1+\rho_{\theta }^{k}\right)}^{4}\left(1-P_{1,\theta }^{k}\right)}\frac{\partial \rho_{\theta }^{k}}{\partial \theta_{i}}\frac{\partial \rho_{\theta }^{k}}{\partial \theta_{j}}\right\}. \end{array}$$

Taking the inverse of $\tilde{I}$ leads to the CRLB matrix ${\tilde{I}}^{-1}$, and the diagonal entries of ${\tilde{I}}^{-1}$ specify the lower bounds on the estimation accuracy of the corresponding parameters.

In (22), the first-order partial derivative of $\rho_{\theta }^{k}$ is calculated as

(23) $$\begin{array}{c} \frac{\partial \rho_{\theta }^{k}}{\partial \theta_{i}}=-\rho_{\theta }^{k}\left[g_{1}\left(d_{k}\right)\frac{\partial d_{k}}{\partial \theta_{i}}+2\Omega \Phi^{-2\Omega }{\left(\alpha_{k}-\varphi \right)}^{2\Omega -1}g_{2}\left(\alpha_{k},\varphi \right)\left(\frac{\partial \alpha_{k}}{\partial \theta_{i}}-\frac{\partial \varphi }{\partial \theta_{i}}\right)\right], \end{array}$$

and $\frac{\partial \rho_{\theta }^{k}}{\partial \theta_{j}}$ can be readily obtained with similar steps. As $\theta_{i},\theta_{j}\in \left\{x,y,\varphi \right\}$, the partial derivatives required in (23) are derived from (4) and (5), i.e.,

(24) $$\begin{array}{ccc} \frac{\partial d_{k}}{\partial x} & = & \frac{x-X_{s}}{\sqrt{{\left(x-X_{s}\right)}^{2}+{\left(y-Y_{s}\right)}^{2}}}+\frac{x-X_{k}}{\sqrt{{\left(x-X_{k}\right)}^{2}+{\left(y-Y_{k}\right)}^{2}}},\\ \frac{\partial d_{k}}{\partial y} & = & \frac{y-Y_{s}}{\sqrt{{\left(x-X_{s}\right)}^{2}+{\left(y-Y_{s}\right)}^{2}}}+\frac{y-Y_{k}}{\sqrt{{\left(x-X_{k}\right)}^{2}+{\left(y-Y_{k}\right)}^{2}}},\frac{\partial d_{k}}{\partial \varphi }=0, \end{array}$$

(25) $$\begin{array}{c} \frac{\partial \alpha_{k}}{\partial x}=\frac{Y_{k}-y}{{\left(x-X_{k}\right)}^{2}+{\left(y-Y_{k}\right)}^{2}},\frac{\partial \alpha_{k}}{\partial y}=\frac{x-X_{k}}{{\left(x-X_{k}\right)}^{2}+{\left(y-Y_{k}\right)}^{2}},\frac{\partial \alpha_{k}}{\partial \varphi }=0, \end{array}$$

and

(26) $$\begin{array}{c} \frac{\partial \varphi }{\partial x}=0,\frac{\partial \varphi }{\partial y}=0,\frac{\partial \varphi }{\partial \varphi }=1. \end{array}$$

Though the GLRT based on one-bit quantized data is easy to implement with only one-bit communication required between sensors and the FC, it suffers severe information loss during observation quantization at sensors which may lead to an unacceptable performance at low signal-noise-ratios (SNRs). To mitigate this degradation, we propose the GLRT detector which employs multi-bit quantization on sensor observations.

## 4. Target Detection and Localization by GLRT Employing an Adaptive Multi-Bit Quantizer

In this section, we consider a GLRT detector based on multi-bit quantization where sensor observations are uniformly quantized. Considering the aspect dependence of the target reflection, an adaptive multi-bit quantizer is proposed to efficiently transmit the observed information from sensors to the FC. Then, the GLRT based on adaptive multi-bit quantization is derived for target detection and localization. Additionally, the CRLB matrix of the MLE is also derived.

### 4.1. Adaptive Multi-Bit Quantization

The optimal multi-bit (or multi-level) quantizer design has been widely studied where the main challenge is the high computational complexity as the design of a multi-level quantizer often involves a nonlinear, multi-dimensional search process [9]. Due to the lack of knowledge of the target parameters, it seems impossible to optimize the quantization strategy. Therefore, it is natural to uniformly quantize the sensor observations, and to employ the same quantization thresholds among all of the sensors.

The typical *Q*-bit uniform quantization scheme quantizes the observations into one of the $M≜2^{Q}$ quantization levels as follows:

(27) $$\begin{array}{c} m_{k}=\left\{\begin{array}{cc} 0, & \;0<r_{k}<\Gamma ,\\ 1, & \;\Gamma \leq r_{k}<2\Gamma ,\\ \vdots \\ M-1, & \;\left(M-1\right)\Gamma \leq r_{k}<\infty , \end{array}\right.,\;k=1,2,\cdots ,K, \end{array}$$

where $m_{k}$ denotes the quantizer output of the *k*-th sensor, and Γ is the quantization parameter. Then, the quantized data ($m_{k}$) is encoded into a binary codeword with fixed number of bits (i.e., *Q* bits) according to a certain encoding rule, before being transmitted to the FC.

In the aspect dependent target reflection model, much of the reflected energy from the target will be concentrated within a particular conical angle (cf. Figure 1). Therefore, when the target is present, only a few sensors located in a certain zone can receive the reflected waves and hence, have relative larger valued observations, while the other sensors merely observe noises with probably small values. On the other hand, it is known from refs. (1) and (2) that the probability of the sensor observation taking a specific value decreases rapidly with a growing $r_{k}$ under both $H_{0}$ and $H_{1}$. Consequently, we can conclude that only those rarely occurring but relatively large valued $\left\{r_{k}\right\}$s, and hence, large quantizer outputs ($\left\{m_{k}\right\}$s) contain information about the target. To strike a balance between using less bits to quantize most of the observations containing probably small valued noise and using more bits to represent those rare but relatively large valued observations containing valuable knowledge about the target, we propose the following adaptive *Q*-bit quantization scheme which quantizes the sensor observations into one of the $L≜2^{Q+1}-2$ quantization levels, i.e.,

(28) $$\begin{array}{c} m_{k}=\left\{\begin{array}{cc} 0, & \;0<r_{k}<\Lambda ,\\ 1, & \;\Lambda \leq r_{k}<2\Lambda ,\\ \vdots \\ L-1, & \;\left(L-1\right)\Lambda \leq r_{k}<\infty , \end{array}\right.,\;k=1,2,\cdots ,K, \end{array}$$

where Λ denotes the quantization parameter of the proposed quantizer. Correspondingly, the quantizer output $m_{k}$ is adaptively encoded into binary codeword $w_{k}$ following the encoding scheme shown in Table 1. The design philosophy of the proposed encoding scheme is described as follows. Different from the conventional encoding scheme that employs fixed number of bits to encode the quantized message, here, the codeword length of $w_{k}$, denoted by $q_{k}$, is determined by the value of $m_{k}$. More specifically, shorter codeword lengths are used to represent smaller $m_{k}$s that have larger occurring probabilities, while longer codeword lengths are employed to represent rarely occurring but larger valued $m_{k}$s. In this encoding manner, each sensor can efficiently transmit its quantized data to the FC, which is important to the WSNs with strictly constrained bandwidths and energy levels. Recall that the number of quantization levels in the typical *Q*-bit quantization scheme is $M=2^{Q}$, while our proposed adaptive *Q*-bit quantizer has more quantization levels available, i.e., $L≜2^{Q+1}-2$. Thus, the proposed quantization scheme achieves much more precise quantization than the typical quantizer, which is another benefit of the proposed scheme.

Note that the choice of the quantization parameter Λ for the proposed quantizer should affect the final detection and localization performance. However, the optimization of parameter Λ is a impermissible task due to the unavailability of target parameters. Nevertheless, we set Λ to be the same as the quantization threshold in the one-bit quantization case described in Section 3, i.e., set Λ=Δ to gain insight into the performance of the detector employing the proposed adaptive quantizer as well as to make a relatively fair comparison with the one-bit quantization-based GLRT.

To be specific, the resulting communication overhead of the proposed adaptive *Q*-bit quantizer is quantitatively analyzed as follows. Denote the expected total number of bits required to be transmitted to the FC under hypotheses $H_{1}$ and $H_{0}$ as $N_{1,\theta }$ and $N_{0}$, respectively. For the proposed quantizer, the pmf of codeword length $q_{k}$ under hypothesis $H_{1}$ is given by

(29) $$\begin{array}{c} \chi_{1,\theta }\left(q_{k}\right)≜\mathrm{Pr}\left(q_{k}|H_{1}\right)=\{\begin{array}{cc} \int_{(2^{q_{k}}-2)\Delta }^{(2^{q_{k}+1}-2)\Delta }f_{1,\theta }^{k}\left(r_{k}\right)dr_{k}={\left(P_{1,\theta }^{k}\right)}^{\left(2^{q_{k}}-2\right)}-{\left(P_{1,\theta }^{k}\right)}^{\left(2^{q_{k}+1}-2\right)}, & 1\leq q_{k}<Q,\\ \int_{(2^{q_{k}}-2)\Delta }^{\infty }f_{1,\theta }^{k}\left(r_{k}\right)dr_{k}={\left(P_{1,\theta }^{k}\right)}^{\left(2^{q_{k}}-2\right)}, & q_{k}=Q, \end{array} \end{array}$$

where the notation $P_{1,\theta }^{k}$ defined in (10) is employed. The corresponding expectation of total number of bits under transmission is then calculated as

(30) $$\begin{array}{c} N_{1,\theta }=\sum_{k=1}^{K}\sum_{q_{k}=1}^{Q}q_{k}\cdot \chi_{1,\theta }\left(q_{k}\right)=\sum_{k=1}^{K}\sum_{q_{k}=1}^{Q}\exp\left[-\frac{(2^{q_{k}}-2)\Delta }{2\eta \sigma^{2}\left(1+\rho_{\theta }^{k}\right)}\right]. \end{array}$$

Similarly, the expected communication overhead between sensors and the FC under hypothesis $H_{0}$ can be derived as

(31) $$\begin{array}{c} N_{0}=\sum_{k=1}^{K}\sum_{q_{k}=1}^{Q}\exp\left[-\frac{(2^{q_{k}}-2)\Delta }{2\eta \sigma^{2}}\right]. \end{array}$$

Compared with the typical *Q*-bit quantization scheme in ref. (27) that has the expected communication overhead between sensors and the FC fixed as Q·K, the proposed adaptive multi-bit quantization scheme greatly reduces the expected communication overhead under both $H_{1}$ and $H_{0}$. Moreover, the expected communication overhead of the proposed quantization scheme increases slowly with a growing *Q*, while the expected communication overhead of the typical multi-bit quantization increases proportionally to *Q*. More specifically, it is shown later by simulation that the adaptive 2- or 3-bit quantizer only incurs a fractional increase of communication overhead compared with the one-bit quantizer in Section 3, and this minor compromise brings substantial performance gain.

It is worth mentioning that the encoded data ${\left\{w_{k}\right\}}_{k=1}^{K}$ with variable codeword length may increase the complexity of data reception at the FC. Nevertheless, we assume that the binary codewords ${\left\{w_{k}\right\}}_{k=1}^{K}$ can be received without errors, with the help of symbol synchronization (e.g., [26]).

### 4.2. Derivation of GLRT Detector

Assume that the encoded data ${\left\{w_{k}\right\}}_{k=1}^{K}$ can be transmitted to the FC reliably. Then, the FC is responsible for making a global decision based on the recovered messages ${\left\{m_{k}\right\}}_{k=1}^{K}$.

For the proposed adaptive *Q*-bit quantization scheme, the probability that the output data ($m_{k}$) takes the specific value *l* under hypothesis $H_{1}$ can be calculated as

(32) $$\begin{array}{c} U_{1,\theta }^{k,l}≜\mathrm{Pr}\left(m_{k}=l|H_{1}\right)=\left\{\begin{array}{cc} {\left(P_{1,\theta }^{k}\right)}^{l}-{\left(P_{1,\theta }^{k}\right)}^{l+1}, & \;0\leq l<L-1,\\ {\left(P_{1,\theta }^{k}\right)}^{l}, & \;l=L-1. \end{array}\right. \end{array}$$

Correspondingly, the pmf of message ($m_{k}$) under $H_{1}$ is

(33) $$\begin{array}{c} s_{1,\theta }^{k}\left(m_{k}\right)=\prod_{l=1}^{L-1}{\left(U_{1,\theta }^{k,l}\right)}^{\delta (m_{k}-l)}, \end{array}$$

where δ(·) denotes the Dirac function. Similarly, the probability that $m_{k}$ takes the value *l* under $H_{0}$ is given by

(34) $$\begin{array}{c} U_{0}^{k,l}≜\mathrm{Pr}\left(m_{k}=l|H_{0}\right)=\left\{\begin{array}{cc} {\left(P_{0}^{k}\right)}^{l}-{\left(P_{0}^{k}\right)}^{l+1}, & \;0\leq l<L-1,\\ {\left(P_{0}^{k}\right)}^{l}, & \;l=L-1, \end{array}\right. \end{array}$$

where the notation $P_{0}$ defined in (9) is employed. The corresponding pmf of $m_{k}$ under $H_{0}$ can be written as

(35) $$\begin{array}{c} s_{0}^{k}\left(m_{k}\right)=\prod_{l=1}^{L-1}{\left(U_{0}^{k,l}\right)}^{\delta (m_{k}-l)}. \end{array}$$

On the basis of the received data, m $≜[m_{1},m_{2},\cdots ,m_{K}]$, the FC is faced to tackle the following composite hypothesis testing problem.

(36) $$\begin{array}{c} \left\{ \begin{array}{cc} H_{0}: & \;m_{k}∼s_{0}^{k}\left(m_{k}\right),k=1,2,\cdots ,K,\\ H_{1}: & \;m_{k}∼s_{1,\theta }^{k}\left(m_{k}\right),k=1,2,\cdots ,K. \end{array}\right. \end{array}$$

Accordingly, the GLRT should be employed to simultaneously reach a final decision and give the target location estimation once it is detected. Similar to ref. (16), the MLE of θ is given by

(37) $$\begin{array}{c} \bar{\theta }≜\arg\max_{\theta }\left[\prod_{k=1}^{K}s_{1,\theta }^{k}\left(m_{k}\right)\right]=\arg\max_{\theta }\sum_{k=1}^{K}\sum_{l=0}^{L-1}\delta (m_{k}-l)\log\left(U_{1,\theta }^{k,l}\right), \end{array}$$

which can also be solved by the DE algorithm. The corresponding GLLR is then computed as

(38) $$\begin{array}{c} \bar{T}\left(m\right)=\log\frac{\max_{\theta }\prod_{k=1}^{K}s_{1,\theta }\left(m_{k}\right)}{\prod_{k=1}^{K}s_{0}^{k}\left(m_{k}\right)}=\sum_{k=1}^{K}\sum_{l=0}^{L-1}\delta \left(m_{k}-l\right)\log\frac{U_{1,\bar{\theta }}^{k,l}}{U_{0}^{k,l}}. \end{array}$$

The final decision is easily made by comparing the GLLR with a predetermined threshold.

### 4.3. Discussion and Derivation of the Cramer–Rao Lower Bound

The global false alarm and detection probability of the GLRT detector based on adaptive multi-bit quantization have similar definitions as in ref. (21); however, neither of these two performance indicators can be analytically derived since the conditional probability density functions of $P(\bar{T}\left(m\right)|H_{0})$ and $P(\bar{T}\left(m\right)|H_{1})$ are not available. The simulation results under hypothesis $H_{0}$ help to find global decision thresholds for the detector, following a constant false alarm probability setup. Later, in Section 5, we present simulation results to reveal the detection performance of the proposed GLRT detector. On the other hand, if the target is detected, the CRLB matrix of the MLEs of target parameters is derived to evaluate the target localization performance.

Denote $\bar{I}$ as the FIM for θ under the adaptive multi-bit quantization scheme which is derived as follows to characterize the CRLB matrix. According to ref. (37), the log-likelihood function of θ can be written as

(39) $$\begin{array}{c} {\bar{L}}_{m}\left(\theta \right)=\log\left[\prod_{k=1}^{K}s_{1,\theta }^{k}\left(m_{k}\right)\right]=\sum_{k=1}^{K}\sum_{l=0}^{L-1}\delta (m_{k}-l)\log\left(U_{1,\theta }^{k,l}\right). \end{array}$$

Taking the second-order partial derivative of (39) with respect to $\theta_{i}$ and $\theta_{j}$, we obtain

(40) $$\begin{array}{c} \frac{\partial^{2}{\bar{L}}_{m}\left(\theta \right)}{\partial \theta_{i}\partial \theta_{j}}=\sum_{k=1}^{K}\sum_{l=0}^{L-1}\left[-\frac{\delta (m_{k}-l)}{{\left(U_{1,\theta }^{k,l}\right)}^{2}}\frac{\partial U_{1,\theta }^{k,l}}{\partial \theta_{i}}\frac{U_{1,\theta }^{k,l}}{\partial \theta_{j}}+\frac{\delta (m_{k}-l)}{U_{1,\theta }^{k,l}}\frac{\partial^{2}U_{1,\theta }^{k,l}}{\partial \theta_{i}\partial \theta_{j}}\right], \end{array}$$

where $\theta_{i},\theta_{j}\in \left\{x,y,\varphi \right\}$. The first-order partial derivative of $P_{1,\theta }^{k}$ required in (40) is written as

(41) $$\begin{array}{c} \frac{\partial U_{1,\theta }^{k,l}}{\partial \theta_{i}}=\{\begin{array}{cc} \left[l{\left(P_{1,\theta }^{k}\right)}^{l-1}-\left(l+1\right){\left(P_{1,\theta }^{k}\right)}^{l}\right]\frac{\partial P_{1,\theta }^{k}}{\partial \theta_{i}}, & 0\leq l<L-1,\\ l{\left(P_{1,\theta }^{k}\right)}^{l-1}\;\frac{\partial P_{1,\theta }^{k}}{\partial \theta_{i}}, & l=L-1, \end{array} \end{array}$$

and the second-order partial derivative of $P_{1,\theta }^{k}$ can be computed as

(42) $$\begin{array}{c} \frac{\partial^{2}U_{1,\theta }^{k,l}}{\partial \theta_{i}\partial \theta_{j}}=\left\{\begin{array}{cc} \;\;\;\left[l\left(l-1\right){\left(P_{1,\theta }^{k}\right)}^{l-2}-l\left(l+1\right){\left(P_{1,\theta }^{k}\right)}^{l-1}\right]\frac{\partial P_{1,\theta }^{k}}{\partial \theta_{i}}\frac{\partial P_{1,\theta }^{k}}{\partial \theta_{j}}\\ +\left[l{\left(P_{1,\theta }^{k}\right)}^{l-1}-\left(l+1\right){\left(P_{1,\theta }^{k}\right)}^{l}\right]\frac{\partial^{2}P_{1,\theta }^{k}}{\partial \theta_{i}\partial \theta_{j}}, & 0\leq l<L-1,\\ l\left(l-1\right)\left[{\left(P_{1,\theta }^{k}\right)}^{l-2}\right]\frac{\partial P_{1,\theta }^{k}}{\partial \theta_{i}}\frac{\partial P_{1,\theta }^{k}}{\partial \theta_{j}}+l{\left(P_{1,\theta }^{k}\right)}^{l-1}\;\frac{\partial^{2}P_{1,\theta }^{k}}{\partial \theta_{i}\partial \theta_{j}}, & l=L-1, \end{array}\right. \end{array}$$

where $\frac{\partial P_{1,\theta }^{k}}{\partial \theta_{i}}=\frac{\Delta P_{1,\theta }^{k}}{2\eta \sigma^{2}{\left(1+\rho_{\theta }^{k}\right)}^{2}}\frac{\partial \rho_{\theta }^{k}}{\partial \theta_{i}}$.

Note that $E_{1,\theta }\left[\delta \left(m_{k}-l\right)\right]=U_{1,\theta }^{k,l}$ and $\sum_{l=0}^{L-1}\frac{\partial^{2}U_{1,\theta }^{k,l}}{\partial \theta_{i}\partial \theta_{j}}=0$. The $\left(i,j\right)$-th element of the FIM is then obtained by taking the negative expectation of (40) with respect to $m_{k}$, i.e.,

(43) $$\begin{array}{c} {\left[\;\bar{I}\;\right]}_{i,j}=-E_{1,\theta }\left[\frac{\partial^{2}{\bar{L}}_{m}\left(\theta \right)}{\partial \theta_{i}\partial \theta_{j}}\right]=\sum_{k=1}^{K}\left\{\frac{\Delta^{2}P_{1,\theta }^{k}\left[1-{\left(P_{1,\theta }^{k}\right)}^{L-1}\right]}{4\eta^{2}\sigma^{4}{\left(1+\rho_{\theta }^{k}\right)}^{4}{\left[1-P_{1,\theta }^{k}\right]}^{2}}\frac{\partial \rho_{\theta }^{k}}{\partial \theta_{i}}\frac{\partial \rho_{\theta }^{k}}{\partial \theta_{j}}\right\}. \end{array}$$

The CRLB matrix is then ${\left[\;\bar{I}\;\right]}^{-1}$. Recalling that $P_{1,\theta }^{k}<1$ and $L=2^{Q+1}-2$, it can be expected that the term $1-{\left(P_{1,\theta }^{k}\right)}^{L-1}$ in (43) will quickly approach 1 with a growing number of quantization bits (*Q*). Therefore, it is enough to use a small number of bits (e.g., 2 or 3 bits) for quantization of the sensor observations in terms of closely achieving the localization performance when L=∞. A further increase in the number of quantization bits brings negligible gain in the localization performance, except for adding a communication burden.

Note that the FIM for the GLRT employing the adaptive multi-bit quantizer (i.e., ref. (43)), as well as the FIM for the GLRT based on one-bit quantized data (i.e., ref. (22)), is a summation of *K* components. The contributions of the *k*-th sensor to the (i,j)-th element of the FIM are denoted as ${\bar{I}}_{i,j}^{k}$ and ${\tilde{I}}_{i,j}^{k}$ for the GLRT detectors based on multi-bit and one-bit quantized data, respectively, i.e.,

(44) $$\begin{array}{ccc} {\bar{I}}_{i,j}^{k} & ≜ & \frac{\Delta^{2}P_{1,\theta }^{k}\left[1-{\left(P_{1,\theta }^{k}\right)}^{L-1}\right]}{4\eta^{2}\sigma^{4}{\left(1+\rho_{\theta }^{k}\right)}^{4}{\left(1-P_{1,\theta }^{k}\right)}^{2}}\frac{\partial \rho_{\theta }^{k}}{\partial \theta_{i}}\frac{\partial \rho_{\theta }^{k}}{\partial \theta_{j}},\\ {\tilde{I}}_{i,j}^{k} & ≜ & \frac{\Delta^{2}P_{1,\theta }^{k}}{4\eta^{2}\sigma^{4}{\left(1+\rho_{\theta }^{k}\right)}^{4}\left(1-P_{1,\theta }^{k}\right)}\frac{\partial \rho_{\theta }^{k}}{\partial \theta_{i}}\frac{\partial \rho_{\theta }^{k}}{\partial \theta_{j}}. \end{array}$$

Consequently, we have the ratio

(45) $$\begin{array}{ccc} \frac{{\bar{I}}_{i,j}^{k}}{{\tilde{I}}_{i,j}^{k}} & = & \frac{1-{\left(P_{1,\theta }^{k}\right)}^{L-1}}{1-P_{1,\theta }^{k}}\geq 1. \end{array}$$

This result agrees with our intuition whereby the GLRT employing the multi-bit quantizer obtains more precise information from sensors, compared with that based on one-bit quantized data. Specifically, the information gain at the *k*-th sensor is determined by the term $\frac{1-{\left(P_{1,\theta }^{k}\right)}^{L-1}}{1-P_{1,\theta }^{k}}$.

## 5. Simulation Results

In this section, we provide simulation results to demonstrate the performance of our proposed GLRT detectors under different settings. The simulations were carried out on a core i5-8250U 3.4 GHz personal computer using MATLAB software. Following refs. [18,20], an active source was assumed to be located at (0,0), and η=1, $\sigma^{2}=1$, Δ=5.9915 was set (thus giving rise to $P_{0}=0.05$). Also, the beampattern parameters of the reflection model were assumed to be Ω=4 and Φ=5π/180. The constant $C_{0}$ was defined through $C_{0}=\gamma_{\mathrm{ref}}\cdot \mathrm{SNR}$, such that the specified SNR was achieved at the reference distance $\gamma_{\mathrm{ref}}$. In our tests, we set $\gamma_{\mathrm{ref}}=1600$. The sensors were deployed within the area {(X,Y)|−1200≤X≤1200, 0≤Y≤240}. Note that although our proposed method works for any sensor deployment pattern as long as sensor locations are known, we assumed uniformly deployed sensors here for simplicity. Specifically, sensors were deployed on the grids of $\left\{\left(X_{k},Y_{k}\right)|X_{k}=a_{x}\cdot D_{\min},Y_{k}=a_{y}\cdot D_{\min}\right\}$, where $a_{x}$, $a_{y}$ are integers, and $D_{\min}$ is the minimum distance between sensors. The experiment time for Monte Carlo (MC) simulations is $10^{6}$. Note that the exact detection and localization performance may be target dependent. Nevertheless, we assumed a target located at (0,800) with the principal reflection angle being φ=π/6 (or 30°), to gain insight into the performance of the proposed schemes.

As mentioned before, the solutions of the MLEs in refs. (16) and (37) used the DE algorithm. The corresponding parameters were set as follows. The population size was $N_{p}=24$, the maximal generation number was $G_{\max}=60$, the scalar number was F=0.5, and the crossover rate was $C_{r}=0.9$. Additionally, the upper and lower bounds for searching the target parameters θ were set as $\theta_{\max}=\left[1000,1300,\frac{\pi }{4}\right]$ and $\theta_{\min}=\left[-1000,300,-\frac{\pi }{4}\right]$, respectively.

### 5.1. Detection Performance Evaluation

We first examined the detection performance of the GLRT based on one-bit quantized data and the GLRT detector employing multi-bit adaptive quantizer under different sensor deployments. Two uniformly deployed sensor fields were considered: (a) a sensor field with $D_{\min}=120$, and thus $-10\leq a_{x}\leq 10$, $0\leq a_{y}\leq 2$, with K=63 sensors; amd (b) a sensor field with $D_{\min}=80$, and thus $-15\leq a_{x}\leq 15$, $0\leq a_{y}\leq 3$, and K=124.

**Figure 2:**
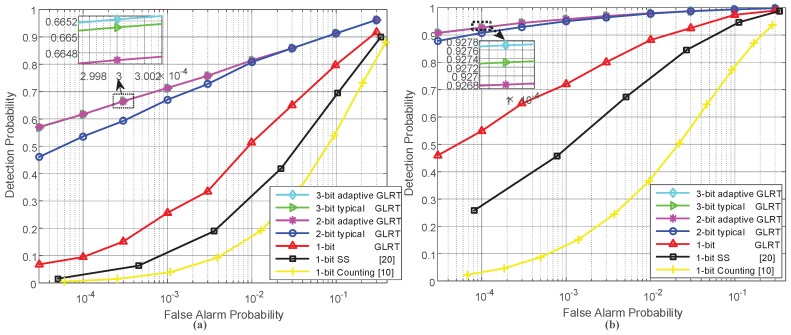
Receiver operating characteristic curves when the signal-noise-ratio (SNR) was 8 dB, and different sensor deployments were employed: (**a**) a sensor field consisting of 3 rows and 21 columns of sensors; (**b**) a sensor field consisting of 4 rows and 31 columns of sensors.

Figure 2 illustrates the receiver operating characteristic (ROC) curves of the proposed GLRT detectors either based on one-bit or adaptive multi-bit quantization under the assumed sensor deployments. The ROC curve of the typical counting rule test based on one-bit data from sensors [10] and that of the decentralized detection scheme proposed in ref. [20], which employs the scan statistic (SS) to fuse the one-bit quantized sensor measurements as well as the ROC curves of the GLRT detectors using typical multi-bit quantizers, are also given for comparison. For the scan statistic-based scheme, its scanning window length is set at 7. Correspondingly, Table 2 shows the expected communication overhead between sensors and the FC for the detectors mentioned above.

We can observe from Figure 2 that the GLRT detectors greatly outperform the existing scan statistic-based scheme and the counting rule test, even with one-bit quantization; this is because more prior knowledge about the target reflection model and the sensor locations is utilized by the GLRT detectors. By combining Figure 2 and Table 2, it is found that the GLRT based on adaptive 2-bit quantization achieves better performance than the GLRT employing the typical 2-bit quantizer at low false alarm probabilities; meanwhile, the communication overhead of the former is significantly reduced. Also, similar phenomenons can be observed for the GLRT detectors either based on adaptive 3-bit quantization or typical 3-bit quantization. These results can be explained as follows. Compared with the typical quantizer with the same number of quantization bits, the adaptive quantizer achieves more precise quantization since it has more quantization levels available; meanwhile, the adaptive quantizer encodes the quantized data into binary codewords more smartly, in the sense of using short codeword lengths to represent the quantized data that have small values but frequently appear. It is also viewed from Figure 2 that the GLRT detectors with 3-bit quantization bring in negligible gain in the detection performance, compared with the adaptive 2-bit quantization-based GLRT. Thus, it is enough to use a small number of bits to quantize sensor observations.

Table 3 specifies the computational time of the above-mentioned detectors with a given deciding threshold (e.g., 8). It is found that the computational time of the scan statistic-based detection scheme is slightly longer than that of the counting test rule, and both of them are significantly shorter than the computational time of all the GLRT detectors. This is because that the GLRT detectors are required to solve the MLE of target parameters and have more complicated expressions for test statistics.

**Figure 3:**
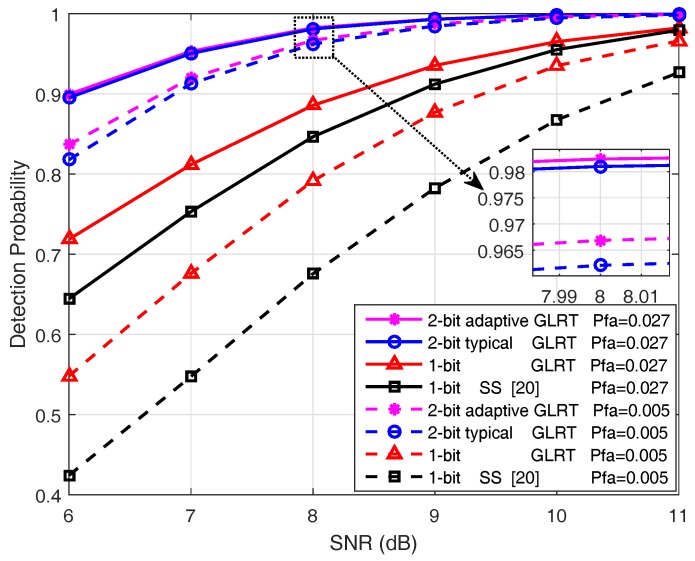
Detection probability versus SNR under fixed false alarm probabilities where the sensor field consists of 4 rows and 31 columns of sensors (i.e., $D_{\min}=80$).

We then tested the detection performance and the communication overhead of the proposed GLRT detectors under different SNR conditions. The sensor field with 4 rows and 31 columns of sensors (i.e., $D_{\min}=80$) was employed. Figure 3 shows the detection probability as a function of reflected signal’s SNR when the false alarm probability is fixed; correspondingly, Table 4 gives the expected communication overheads of the detectors.

Figure 3 again verifies the behaviors for which the GLRT detector with one-bit quantization outperforms the existing scan statistic-based scheme, and the GLRT employing adaptive 2-bit quantizer achieves better detection performance than the GLRT using a typical 2-bit quantizer, especially at low SNRs. It is noticed from Table 4 that the expected communication overhead of the adaptive 2-bit quantization scheme under $H_{1}$ increases with growing SNR, which agrees with our intuition that more encoded data exists with long codeword lengths at higher SNRs. Nevertheless, the communication burden of the GLRT employing the proposed adaptive 2-bit quantization is still much lower than that using a typical 2-bit quantizer. By combining Figure 3 and Table 4, it is found that the proposed 2-bit quantizer only incurs a fractional increase of communication overhead, compared with the one-bit quantization scheme. Moreover, this minor compromise brings substantial performance gain.

### 5.2. Localization Performance Evaluation

In this subsection, we are interested in the target localization performance of the proposed GLRT detectors. Similar to ref. [18], the root-mean-square-error (RMSE) is used as the localization performance metric. The definition of RMSE is given by

(46) $$\begin{array}{c} \mathrm{RMSE}≜\sqrt{E\left[{\left(\tilde{x}-x\right)}^{2}+{\left(\tilde{y}-y\right)}^{2}\right]}, \end{array}$$

where $\left(\tilde{x},\tilde{y}\right)$ is the MLE of the target location. The lower bounds of the RMSE for different GLRT detectors are then specified by their CRLB matrixes. Specifically, $\sqrt{{\left[\;{\tilde{I}}^{-1}\;\right]}_{1,1}+{\left[\;{\tilde{I}}^{-1}\;\right]}_{2,2}}\;$ gives the lower bound of the RMSE for one-bit quantization based GLRT, and the lower bounds of the RMSE for the GLRT detectors based on 2-bit quantization can be obtained similarly.

**Figure 4:**
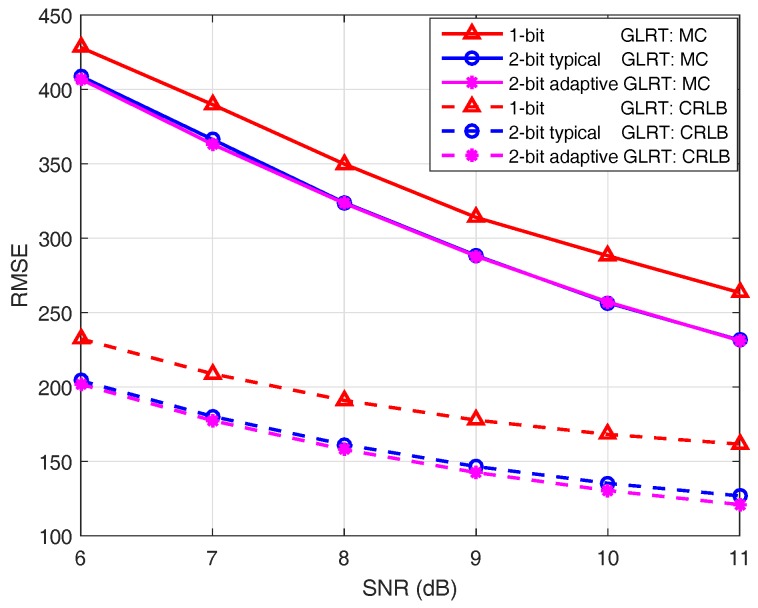
Root-mean-square-error (RMSE) under different SNR conditions where the sensor field consists of 4 rows and 31 columns of sensors.

Figure 4 illustrates the RMSE of the MLE of target location under different SNR conditions. Solid lines are the results of MC experiments; dashed lines are the CRLBs calculated according to the derived expressions (cf. (22) and (43)). It is known from Figure 4 that the RMSEs of MC experiments do not reach their CRLBs. The reason for this is that the MLE is asymptotically efficient when a sufficiently large number of samples are available. While for the aspect dependent target reflection model discussed here, only a few sensor observations are utilized for the maximum likelihood estimation, since the power of the reflected signal is concentrated within a narrow conical angle. It is also observed that the GLRT employing an adaptive 2-bit quantizer achieves much better localization performance than the GLRT based on one-bit quantization at the cost of only a minor increase of communication overhead (cf. Table 4). For all three GLRT detectors, the RMSEs from the MC experiment are shown to be closer to the corresponding CRLBs of RMSE as the SNR grows.

**Figure 5:**
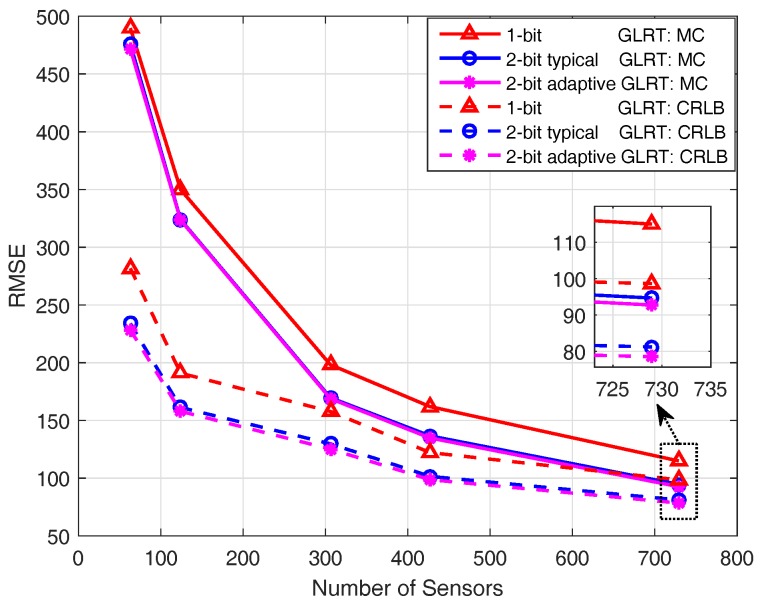
RMSE under various sensor deployments, where SNR = 8 dB.

Figure 5 demonstrates the RMSE under various sensor deployments. More specifically, the minimum distances between sensors are $D_{\min}=120$, 80, 48, 40, and 30, respectively, thus giving rise to the sensor fields with K=63, 124, 306, 427, and 729 sensors. It is observed that when the number of sensors increases, the RMSE decreases considerably; meanwhile, the gap between the RMSE of MC experiments and its CRLB also decreases.

## 6. Conclusions

This paper considered the aspect-aware target detection and localization in a WSN, where the analysis focused on the aspect and distance dependent target reflection model. First, the GLRT detector based on one-bit quantization was proposed to simultaneously achieve target detection and localization. We then proposed the GLRT detector employing an adaptive multi-bit quantizer which quantizes sensor observations more precisely to further improve the detection and localization performance. When the target was detected, the CRLB matrix of the MLE was also derived for the proposed GLRT detector. The numerical results showed that the proposed GLRT detector based on one-bit quantization outperforms the existing scheme that based on scan statistic. Moreover, the proposed GLRT detector employing a adaptive 2-bit quantizer achieves much better performance than the one-bit quantization based GLRT at the cost of only a minor increase in communication overhead.

## Figures and Tables

**Table 1:** Proposed Encoding Scheme.

$m_{k}$	0	1	2	3	4	5	⋯	$2^{Q}-2$	$2^{Q}-1$	⋯	$2^{Q+1}-3$
$q_{k}$	1		2				⋯	*Q*			
$w_{k}$	0	1	00	01	10	11	⋯	$\underset{Q}{\underset{︸}{00\cdots 00}}$	$\underset{Q-1}{\underset{︸}{00\cdots 0}}1$	⋯	$\underset{Q}{\underset{︸}{11\cdots 11}}$

Note: $m_{k}$, $q_{k}$, and $w_{k}$ denote the input message, codeword length, and output codeword, respectively.

**Table 2:** Expected communication overhead under different sensor deployments (SNR = 8 dB).

Sensor Field	Field (a): 3×21					Field (b): 4×31				
Schemes	1-bit GLRT/ SS/Counting	2-bit Adaptive	2-bit Typical	3-bit Adaptive	3-bit Typical	1-bit GLRT/ SS/Counting	2-bit Adaptive	2-bit Typical	3-bit Adaptive	3-bit Typical
Overhead under $H_{1}$	63	64.78	126	65.05	189	124	127.77	248	128.35	372
Overhead under $H_{0}$	63	63.16	126	63.16	189	124	124.31	248	124.31	372

**Table 3:** Computational time of different detection schemes for $10^{6}$ Monte Carlo runs (Unit: Seconds).

	Counting	SS	1-bit GLRT	2-bit Adaptive	2-bit Typical	3-bit Adaptive	3-bit Typical
Field (a): 3×21	1728.6	1746.7	79,581	87,643	86,887	87,632	89,267
Field (b): 4×31	1752.8	2574.1	126,189	136,617	136,390	136,146	134,108

**Table 4:** Expected communication overhead under different SNRs, where $D_{\min}=80$.

SNR (dB)	6	7	8	9	10	11
2-bit adaptive: $H_{1}$	126.58	127.15	127.77	128.42	129.08	129.74
2-bit adaptive: $H_{0}$	124.31					
1-bit GLRT/Scan statistic: $H_{1}$ & $H_{0}$	124					
2-bit typical: $H_{1}$&$H_{0}$	248					
